# Scenario analysis on co-benefits of air pollution control and carbon reduction in Yangtze River Delta based on STIRPAT model

**DOI:** 10.1371/journal.pone.0296915

**Published:** 2024-01-11

**Authors:** Peijiong Feng, Yan Gu, Yaguai Yu, Yinzi Bao, Ruiyan Gao, Taohan Ni

**Affiliations:** 1 School of Economics and Management, Tongji University, Shanghai, China; 2 College of Business and Economics, Australian National University, Canberra, Australia; 3 Business School, Ningbo University, Ningbo, China; 4 Donghai Academy, Ningbo University, Ningbo, China; 5 Business School, University of Nottingham, Ningbo, China; Chongqing University, CHINA

## Abstract

In a changing climate, it is vital to focus on the co-benefits of the pollution control and carbon emission reduction. Based on calculation of emission equivalent, the synergy coefficient is further calculated to quantitatively analyze the co-benefits of air pollution control and carbon reduction in the Yangtze River Delta; Scenario analysis in co-benefits in the Yangtze River Delta from 2026–2035 is thoroughly proposed after STIRPAT model is designed based on influencing factors confirmation including population size, economic scale, industrialization level, urbanization rate and energy intensity from measuring dimensions of synergy coefficient. The results show that the Yangtze River Delta region can partially achieve synergistic emission reduction by 2026 and realize comprehensive synergistic emission reduction of air pollution and carbon emissions not late than 2030, which provides a reference for promoting the decision-making of the new stage of long-term carbon and pollution reduction, and further, realizing carbon peak regulation and carbon neutrality.

## 1. Introduction

With countries’ ongoing investigation of global climate regulation, the objective of carbon neutrality has given birth to a new phase of global green and low-carbon growth. More than 130 countries and regions, including China, the United States, and Europe, have proposed long-term carbon-neutral emission reduction targets by April 2022 [1]. To actively combat global climate change, China, the world’s largest developing country, has set the strategic targets of reaching a peak in carbon dioxide emissions by 2030 and carbon neutrality by 2060, this also means that China’s environmental pollution prevention and control is facing a new challenge in the synergistic promotion of pollution control and carbon reduction.

From the holistic perspective, China has achieved significant accomplishments in environmental pollution control and carbon reduction in past years. According to Ministry of Ecology and Environment of People’s Republic of China (www.mee.gov.cn accessed in Nov 2023), the policies and measures implemented by China since 2005 to combat climate change have resulted in a saving of 2.21 billion tons of standard coal, as well as significant reductions in sulfur dioxide emissions (11.92 million tons) and nitrogen oxide emissions (11.3 million tons). From the regional perspective, the Yangtze River Delta’s impact on carbon emissions was the most significant among Beijing-Tianjin-Hebei, the Yangtze River Delta, and the Greater Bay Area (Guangdong) from 2015 to 2019. The Yangtze River Delta saw the largest decrease in the amount of carbon emissions, down to 19.65%; however, the three regions all saw an increase in the amount of carbon emissions per person, with the Yangtze River Delta experiencing the smallest increase at 3.06 percent (www.greenpeace.org.cn accessed in Nov 2023).

Nevertheless, that is just a fresh beginning rather than the end. The Yangtze River Delta may continue to undertake the "double carbon" action in this region, spread and encourage the green and low-carbon transformation in the surrounding areas, and improve regional coordination based on the successful low-carbon transformation over the past 15 years. In light of this, this paper selects three provinces and one city in the Yangtze River Delta(hereinafter referred to as “three provinces and one city”) as the research object, quantitatively analysing the Co-benefits in this region under various scenarios based on data related to air pollution and greenhouse gas emissions from 2012 to 2021, hoping to offer guidance for promoting the Co-benefits of air pollution control and carbon reduction(hereinafter referred to as “Co-benefits”).

## 2. Literature review

Co-benefits are derived from ancillary benefits, which are presented to demonstrate how actions reduce carbon emissions can also have a positive impact on the reduction of other pollutants [2]. The terms "Co-benefits" were first introduced by the Intergovernmental Panel on Climate Change in its third scientific assessment reports on climate change [3] and has been widely used to describe the occurrence whereby taking action to reduce pollution emissions results in additional environmental advantages [4].

Currently, studies on the research methods and research area of the co-benefits of environmental pollution and greenhouse gas emissions are relatively comprehensive worldwide. From the perspective of research methods, the following methods has been widely adopted in assessing the cross-synergy between co-benefits and policies as well as economy: Computable General Equilibrium (CGE) Model [5–7], Correlation Coefficient Method [8, 9], Synergy Evaluation Index Method [10], Panel Regression Model [11, 12], Scenario Analysis Method [10, 13], Multivariate Linear Regression Analysis Method [9] and also, Stochastic Impacts by Regression on Population, Affluence, and Technology (STIRPAT) Model [9]. As far as STIRPAT model method is concerned, currently 1634 academic journals have adopted this model to conduct quantitative analysis on the influencing factors of carbon emissions, and it has been mostly used for regional peak carbon dioxide emissions prediction in the latest five years. From the perspective of research area in China, the research areas covered the national level [14, 15], the regional level [10, 16, 17] and the level of a single province and city [8, 9, 18–20].

Undoubtedly, a number of studies are conducted to single city or specific section. Few papers discussed the pertinent research on the co-benefits in the Yangtze River Delta, particularly the variations between provinces and cities under different scenarios. Accordingly, this paper selects the Yangtze River Delta as the research area, adopts the emission factor method to account for the air pollution emissions, and calculates the synergy coefficient based on Tapio-decoupling Model; Further, constructs STIRPAT Model of co-benefits based on the relevant data of air pollution and carbon emissions from 2012 to 2021, and the scenario analysis of the synergistic emission equivalent and synergy coefficient in the Yangtze River Delta is carried out in two scenarios: baseline and low carbon, in order to provide meaningful recommendations for the Yangtze River Delta region to achieve sustainable synergy based on the co-benefits of pollution reduction and carbon reduction.

## 3. Research methods and data sources

### 3.1 Data processing

#### 3.1.1 Measurement of emission equivalent of air pollution

In this paper, "pollution" in reducing pollution and carbon refers to air pollution in environmental pollutants. The results of spatial analysis show that the areas with serious air pollution are highly overlapped with the key areas of carbon dioxide emissions [21]. Considering that it is difficult to make a unified comparative analysis of water, gas, soil and solid waste, this paper only selects air pollution to calculate. The first one is the emission of air pollution, which is calculated by referring to the method of He Kebin et al. [22]. As shown in Formula (1):

$$E=EF\times A\times \left(1-\eta \right)$$ (1)

In Formula (1), *E* is the emission of air pollution, *EF* is the pollutant production coefficient, *A* is the fuel consumption or product output or the activity level or administrative areas, and *η* is the pollutant removal efficiency of pollution control facilities.

Due to the wide variety of air pollution, this paper converts the emissions of each air pollutant into the emission equivalent for accounting. The definition of air pollutant emission equivalent is derived from the Implementation Regulations of People’s Republic of China (PRC) Environmental Protection Tax Law. Use Formula (2) to calculate the equivalent emission of air pollution.


$$E_{LAP}=\alpha E_{SO_{2}}+\beta E_{NO_{x}}+\gamma E_{CO}+\delta E_{VOC_{S}}+\varepsilon E_{NH_{3}}+\zeta E_{PM_{10}}$$ (2)

In this paper, the emission equivalent of air pollution is calculated based on the emission of air pollution (Formula 1). In Formula (2), *E*_*LAP*_ is the equivalent of air pollution in tons (t); $E_{SO_{2}}$ is the emissions of *SO*_2_ in tons,(t); $E_{NO_{x}}$ is the emissions of *NO*_*x*_ in tons (t); *E*_*CO*_ is the emissions of *CO* in tons(t); $E_{VOC_{S}}$ is the emissions of *VOC*_*S*_ in tons(t); $E_{NH_{3}}$ is the emissions of *NH*_3_ in tons (t); $E_{PM_{10}}$ is the emissions of *PM*_10_ in tons (t); *α*, *β*, *γ*, *δ*, *ε* and *ζ* are the equivalent coefficients of *SO*_2_, *NO*_*x*_, *CO*, *VOC*_*S*_, *NH*_3_ and *PM*_10_ respectively, dimensionless.

#### 3.1.2 Relevant data sources

Carbon emissions in this paper are taken from ‘China provincial CO_2_ emission inventory’ published by Carbon Emissions Accounts & Datasets (https://www.ceads.net/data/province/ accessed in Nov 2023) which employs apparent emissions accounting method, and the details can be found in paper of Shan et al. [23]. The air pollutant emission data comes from the China Environmental Statistics Yearbook.

The total population, per capita GDP, urbanization rate of permanent residents, energy intensity, industrialization level and other relevant data required for the model construction come from statistical bulletins on national economic and social development and statistical yearbooks of corresponding cities over the years.

### 3.2 Calculation of synergistic emission equivalent and synergy coefficient

#### 3.2.1 Synergistic emission equivalent of air pollution control and carbon reduction

Based on the research results of Liu Maohui et al. [9]. This paper considers the emissions of air pollution and greenhouse gases in a comprehensive way with the synergistic emission equivalent of reducing pollution and reducing carbon (hereinafter referred to as the synergistic emission equivalent), and defines the calculation Formula (3) as follows:

$$Q=\theta E_{LAP}+\kappa E_{GHG}$$ (3)

In Formula (3), Q is the equivalent of synergistic emission in tons (t); *E*_*LAP*_ is the equivalent of air pollution in tons (t); *E*_*GHG*_ is the greenhouse gas emission in tons (t); *θ* and *κ* are equivalent coefficients of air pollution and greenhouse gases respectively, dimensionless. The specific value of equivalent is shown in Table 1.

**Table 1:** Coefficient table of air pollutant and co-emission equivalent.

Type	Equivalent Coefficient	Value	Type	Equivalent Coefficient	Value
SO_2_	*α*	1/0.95	NH_3_	*ε*	1/9.09
NO_x_	*β*	1/0.95	PM_10_	*ζ*	1/2.18
CO	*γ*	1/16.7	***E***_***LAP***_	*θ*	1
VOC_S_	*δ*	1/0.95	***E***_***GHG***_	*κ*	0.00372

Note: The equivalent coefficient values of *SO*_*2*_, *NO*_*x*_, *CO*, *NH*_*3*_ and *PM*_*10*_ are from [https://www.gov.cn/zhengce/content/2017-12/30/content_5251797.htm], *VOC*_*S*_ ’s are from [http://www.mof.gov.cn/gp/xxgkml/szs/201506/t20150626_2510508.htm], and the equivalent coefficient values of air pollution and greenhouse gases are from reference [24].

#### 3.2.2 Synergy coefficient of pollution control and carbon reduction

The theory of "decoupling" is widely used to evaluate the process of resource and environment utilization, and it is usually used to describe the degree and direction of "decoupling". Generally, the total GDP is used as the driving variable, and energy consumption or carbon emissions are used as the explanatory variable [25]. The main methods to measure the decoupling relationship are the elasticity coefficient proposed by Tapio [26] and the decoupling factor [27], the former can eliminate the error in the section of based period better than the latter [16]. Tapio’s elastic analysis method is often used to measure the decoupling relationship between carbon emissions and economic growth. Referring to the expression of Tapio decoupling model, this paper defines the synergy coefficient of air pollution control and carbon reduction (hereinafter referred to as the synergy coefficient): the coefficient to measure the contribution of different control measures to the synergistic control of greenhouse gases and conventional air pollution, so as to quantitatively describe the co-benefits. The synergy coefficient is defined as Formula (4):

$$S=\frac{\Delta E_{LAP}/E_{LAP}}{\Delta E_{GHG}/E_{GHG}}$$ (4)

In Formula (4), S is the synergistic coefficient of reducing pollution and carbon, dimensionless; ΔE_LAP_ stands for the reductions of air pollutant emissions in this year compared with the previous year, in tons (t); E_LAP_ stands for the air pollutant emission in the previous year, and the unit is tons (t); ΔE_GHG_ stands for the reduction of greenhouse gas emission in this year compared with the previous year in tons (t); E_GHG_ stands for the greenhouse gas emissions of the previous year; According to the value range of ΔE_LAP_, ΔE_GHG_ and synergy coefficient S, the characteristics of synergy state can be divided into eight categories (Liu Maohui et al. 2022) [9], as shown in Table 2.

**Table 2:** Division of state characteristics of the co-benefit status.

Synergy State	Δ*E*_*LAP*_	Δ*E*_*GHG*_	*S*	Characteristics
Synergistic emission reduction	< 0	< 0	[0,0.8)	Both are reduced. Δ*E*_*LAP*_ reduces slower than Δ*E*_*GHG*_
	< 0	< 0	[0.8,1.2]	Both are reduced. The rates of both are equal.
	< 0	< 0	(1.2, + *∞*)	Both are reduced. Δ*E*_*LAP*_ reduces faster than Δ*E*_*GHG*_
Synergistic emission increase	> 0	> 0	[0,0.8)	Both are increased. Δ*E*_*LAP*_ increases slower than Δ*E*_*GHG*_
	> 0	> 0	[0.8,1.2]	Both are increased at the same rates
	> 0	> 0	(1.2, + *∞*)	Both are increased. Δ*E*_*LAP*_ increases faster than Δ*E*_*GHG*_
Incompatible	< 0	> 0	(-*∞*, 0)	Reduction of Δ*E*_*LAP*_, Increase of Δ*E*_*GHG*_
	> 0	< 0	(-*∞*, 0)	increase of Δ*E*_*LAP*_, Reduction of Δ*E*_*GHG*_

### 3.3 STIRPAT model of co-benefits of pollution control and carbon reduction

#### 3.3.1 STIRPAT model

Potential carbon dioxide emissions from different regions in the future will be influenced by a series of socioeconomic factors. Therefore, scenario analysis is widely used to study the future trend of carbon emissions, especially scenario analysis based on STIRPAT model. The IPAT model [28] is relatively simple in dealing with the relationship between influencing factors and environmental impact, and it is difficult to reflect the changes of environmental impact effectively and comprehensively. However, as an effective method to quantitatively analyse the environmental loads caused by various influencing factors, STIRPAT model has been widely used in the study of air pollution and greenhouse gas emissions because of its good flexibility and certain expansion space.

The STIRPAT model is derived from the IPAT equation, which is expressed as:

I=P×A×T (5)

In Formula (5), I is the environmental load, P is the population size, A is the wealth, and T is the level of science and technology.

To overcome the deficiency that all factor in the IPAT equation have equal influence on environmental load, Dietz T and Rosa E A [29] constructed the STIRPAT model based on the IPAT equation, and its expression is as follows:

$$I=a\times P^{b}\times A^{c}\times T^{d}\times e$$ (6)

In Formula (6), *a* is a constant, and the meanings of P, A and T are equivalent to Formula (5). *b*, *c* and *d* are exponential terms of P, A and T, and *e* is a random error term.

#### 3.3.2 Factors selection of STIRPAT model

This study retains the original variables (population size and economic scale) of STIRPAT model. Referring to the research conducted by Jiang Huiqin et al. [30] and Li Zhiqing et al. [31], Industrialization level, urbanization level and Energy intensity are vital factors affecting the Co-benefits in the Yangtze River Delta in recent trends. Therefore, the finally chosen five variables as shown in Table 3.

**Table 3:** Description of each variable in the STIRPAT model.

Symbol	Variable	Description	Unit
E	Amount of emissions	Δ*E*_*LAP*_ or Δ*E*_*GHG*_	Tons
A	Economics scale	Per capita GDP	Ten thousand yuan
P	Population size	(year-end) resident population	Ten thousand people
IS	Industrialization level	Ratio of industrial added value to GDP	%
UR	Urbanization level	urban population / total population	%
T	Energy intensity	Energy consumption per unit of GDP	Tons of standard coal/ten thousand yuan

#### 3.3.3 STIRPAT model for co-benefits

Based on the selected influencing factors of the co-benefits in the Yangtze River Delta, the extended STIRPAT model of the influencing factors of emissions is constructed (Formula 7):

$$E=\alpha \times A^{b}\times P^{c}\times IS^{d}\times UR^{f}\times T^{h}\times \varepsilon$$ (7)

In Formula (7), *α* is a constant term, *b*, *c*, *d*, *f*, *g* and *h* are exponential terms and ***ε*** is an error term.

To eliminate the influence of heteroscedasticity that may exist in the model, all variables are logarithmically transformed. The extended STIRPAT model after logarithmic transformation is shown as Formula (8):

$$\text{ln}\;E=\text{ln}\;\alpha +\alpha_{1}\;\text{ln}\;A+\alpha_{2}\;\text{ln}\;P+\alpha_{3}\;\text{ln}\;IS+\alpha_{4}\;\text{ln}\;UR+\alpha_{5}\;\text{ln}\;T+\text{ln}\;\varepsilon$$ (8)

In Formula (8), ***α***_**1**_, ***α***_**2**_, ***α***_**3**_, ***α***_**4**_, ***α***_**5**_ are elastic coefficients, that is to say, when A, P, IS, UR, T change by 1%, they will cause ***α***_**1**_%, ***α***_**2**_%, ***α***_**3**_%, ***α***_**4**_% and ***α***_**5**_% changes of E.

When the STIRPAT model is used to study the emission of air pollution or greenhouse gas, the multiple linear regression model constructed is prone to multiple linear problems. To solve this problem, this study refers to the method of Zhang Zhe et al. [32] and adopts partial least square method (PLS) to construct the multiple linear regression model.

### 3.4 Scenario prediction of STIRPAT model in Yangtze River Delta

#### 3.4.1 The 2022–2025 Numerical values of the STIRPAT model in Yangtze River Delta

According to the Outline of Regional Integration Development Plan for the Yangtze River Delta issued by the Central Committee of the Communist Party of China and the State Council in December 2019, the research area of this paper is the Yangtze River Delta, including Shanghai, Jiangsu, Zhejiang, and Anhui.

The quantitative boundary conditions of the STIRPAT model in the Yangtze River Delta from 2022 to 2025 are basically determined in the 14th Five-Year Plan and the Outline of Vision Goals for 2035 issued by the governments, this paper collates and calculates the 2022–2025 indicators of the STIRPAT model in three provinces and one city (Table 4). In addition, this study assumes that all indicators keep a constant change rate in 2022–2025, based on the actual value in 2021 and the preset indicators in 2025 in the 14th Five-Year Plan.

**Table 4:** Indicators of the STIRPAT model in Yangtze River Delta in 2020–2025.

Indicators by 2025	Shanghai	Jiangsu	Zhejiang	Anhui
A	210,000 yuan	150,000 yuan	130,000 yuan	90,000 yuan
P	25million(2035)	90million (2035)	74million(2035)	61.8million (2025)
IS	25%	40%	33.3%	33.3%
UR	90%	75%	80% (2035)	64%
T (Accumulated reduction)	-14%	-13.5%	-15%	-14.5%

Notes: 1. The population size of Jiangsu Province and Zhejiang Province is based on the Master Plan of Land and Space (2021–2035).

#### 3.4.2 Scenario analysis of STIRPAT model in Yangtze River Delta from 2026 to 2035

The prediction and analysis of the STIRPAT model in the Yangtze River Delta from 2020 to 2035 includes three aspects: (1) setting the main economic and social development indicators proposed by the government in 2025 as specific quantitative boundary conditions; (2) according to the historical development trend, six variables, such as population scale and economic scale, are reasonably assumed, and some internal parameters of the forecasting structure are obtained; (3) according to the historical growth trend of air pollution and carbon emissions and the current social development, two forecasting scenarios are set, namely, baseline scenario and low-carbon scenario.

The boundary conditions of the scenario forecasts are partially determined, and the rest of the parameters under the baseline and the low-carbon scenario for 2026–2035 of three provinces and one city are set out in this paper. The baseline scenario fully refers to the relevant climate change planning policy documents of province and city. In the low-carbon scenario, the parameter setting will be lower than that in the baseline scenario, the population control, energy intensity and industrialization level will be more stringent. Urbanization will proceed more slowly. According to Zhang Chewei, China will reach a "turning point" during the "14^th^ Five-Year Plan" period where rapid urbanization will gradually slow down. Urbanization will continue to slow down up until 2035 during the "14th Five-Year Plan" period; following that, it will move into a rather stable stage. Table 5 displays the precise values for each scenario’s next parameter.

**Table 5:** Forecast parameter setting for cities in Yangtze River Delta in 2026–2035.

Scenario	Parameter	2026–2030 Y				2031–2035 Y			
		Shanghai	Jiangsu	Zhejiang	Anhui	Shanghai	Jiangsu	Zhejiang	Anhui
Baseline scenario	P	0.2	0.3	0.95	0.3	0.1	0.2	0.85	0.2
	A	5.0	4.5	4.5	4.0	4.8	4.3	4.3	3.8
	UR	0.2	0.6	0.7	1.3	0.1	0.5	0.6	1.2
	IS	0.8	0.3	0.7	0.7	0.6	0.2	0.5	0.6
	T	(18)	(16)	(18)	(18)	(20)	(18)	(20)	(20)
Low-carbon scenario	P	0.0	0.2	0.85	0.2	(0.1)	0.1	0.75	0.1
	A	4.8	4.3	4.3	3.8	4.6	4.1	4.1	3.6
	UR	0.1	0.5	0.6	1.2	0.0	0.4	0.5	1.1
	IS	0.6	0.2	0.5	0.5	0.4	0.1	0.3	0.4
	T	(20)	(18)	(20)	(20)	(22)	(20)	(22)	(22)

Note: (1) Units in this table are all percentage; (2)The data corresponding to population size, economic scale, industrialization level, urbanization rate in the two scenarios in Table 5 are the percentage of annual increase (decrease), while data corresponding to energy intensity is the percentage of cumulative decrease from 2020 to 2025.

## 4. Results

### 4.1 Construction and analysis of forecast model

#### 4.1.1 Forecast model in Shanghai

Based on Formula (8) and the data from 2012 to 2021, the prediction model of air pollution and carbon emissions in Shanghai is constructed as follows by using partial least square method.


$$E_{GHG}=EXP\;(-34.158+1.58\;ln\;P+1.023\;ln\;A+8.511\;ln\;UR+0.471\;ln\;IS+1.487\;ln\;T)$$ (9)

$$E_{LAP}=EXP\;(-176.954+3.451\;ln\;P-5.383\;ln\;A+38.085\;ln\;UR+1.25\;ln\;IS-1.682\;ln\;T)$$ (10)

According to the forecast model, with other variables held constant, the air pollutant emission in Shanghai are directly proportional to the population size, urbanization level and industrialization level, for every 1% increase in population size, urbanization level and industrialization level, air pollutant emissions will be increased by a mean of 3.451%,38.085% and 1.25%, respectively, and inversely proportional to the economic scale and energy intensity, for every 1% increase in economic scale and energy intensity, air pollutant emissions will be decreased by a mean of 5.383% and 1.682%. Carbon emissions in Shanghai is directly proportional to all five factors, for every 1% increase in population size, economic scale, urbanization level, industrialization level and energy intensity, carbon emissions will be increased by a mean of 1.58%, 1.023%, 8.511%, 0.471% and 1.487%, respectively.

**Fig 1:**
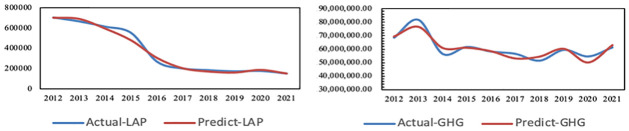
Actual and simulated emissions of air pollution and greenhouse gases in Shanghai from 2012 to 2021.

It is concluded that the adjusted R^2^ of air pollution is 0.968, F = 302.839, p = 0.000<0.05 and the adjusted R^2^ of greenhouse gas is 0.673, F = 6.421, p = 0.048<0.05. Therefore, the prediction model of air pollution and carbon emissions has high precision and can be used for prediction and analysis. Additionally, the results of actual and simulated emissions of air pollution and greenhouse gas are shown in Fig 1.

#### 4.1.2 Forecast model in Jiangsu

Based on Formula (8) and the data from 2012 to 2021, the prediction model of air pollution and carbon emissions in Jiangsu Province is constructed as follows by using partial least square method.


$$E_{GHG}=EXP\;(10.291+1.291\;ln\;P+0.753\;ln\;A-0.388\;ln\;UR-0.25\;ln\;IS+0.868\;ln\;T)$$ (11)

$$E_{LAP}=EXP\;(-362.476+45.836\;ln\;P-4.267\;ln\;A-8.105\;ln\;UR+1.365\;ln\;IS-1.889\;ln\;T)$$ (12)

According to the forecast model, with other variables held constant, air pollutant emission in Jiangsu is directly proportional to the population size and industrialization level, for every 1% increase in population size and industrialization level, air pollutant emissions will be increased by a mean of 45.836% and 1.365% respectively, and inversely proportional to the economic scale, urbanization level and energy intensity, for every 1% increase in economic scale, urbanization level and energy intensity, air pollutant emissions will be decreased by a mean of 4.267%, 8.105% and 1.889%. The Carbon emissions in Jiangsu is directly proportional to the population size, economic scale and energy intensity, for every 1% increase in population size, economic scale and energy intensity, carbon emissions will be increased by a mean of 1.291%, 0.753% and 0.868% respectively, and inversely proportional to the urbanization level and industrialization level, for every 1% increase in urbanization level and industrialization level, carbon emissions will be decreased by a mean of 0.388% and 0.25%.

**Fig 2:**
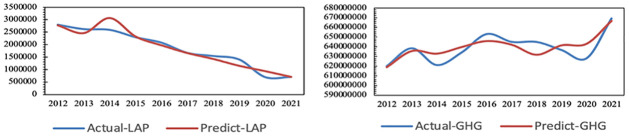
Actual and simulated emissions of air pollution and greenhouse gases in Jiangsu from 2012 to 2021.

It is concluded that the adjusted R^2^ of air pollutant is 0.841, F = 324.431, p = 0.000<0.05 and the adjusted R^2^ of carbon emissions is 0.664, F = 12.429, p = 0.015<0.05. Therefore, the prediction model of air pollution and carbon emissions have high precision and can be used for prediction and analysis. Additionally, the results of actual and simulated emissions of air pollution and greenhouse gas are shown in Fig 2.

#### 4.1.3 Forecast model in Zhejiang

Based on Formula (8) and the data from 2012 to 2021, the prediction model of air pollution and carbon emissions in Zhejiang Province is constructed as follows by using partial least square method.


$$E_{GHG}=EXP\;(45.785-3.322\;ln\;P+2.11\;ln\;A+0.181\;ln\;UR-0.213\;ln\;IS+1.642\;ln\;T)$$ (13)

$$E_{LAP}=EXP\;(47.771-0.331\;ln\;P-0.686\;ln\;A-5.594\;ln\;UR-1.43\;ln\;IS+1.073\;ln\;T)$$ (14)

According to the forecast model, with other variables held constant, air pollutant emission in Zhejiang is directly proportional to the energy intensity, for every 1% increase in energy intensity, air pollutant emission will be increased by a mean of 1.073%, and inversely proportional to other factors, for every 1% increase in population size, economic scale, urbanization level and industrialization level, air pollutant emissions will be decreased by a mean of 0.331%, 0.686%, 5.594% and 1.43%, respectively. The carbon emissions in Zhejiang is directly proportional to the economic scale, urbanization level and energy intensity, for every 1% increase in economic scale, urbanization level and energy intensity, carbon emissions will be increased by a mean of 2.11%, 0.181% and 1.642% respectively, and inversely proportional to the population size and industrialization level, for every 1% increase in population size and industrialization level, carbon emissions will be decreased by a mean of 3.322% and 0.213%.

**Fig 3:**
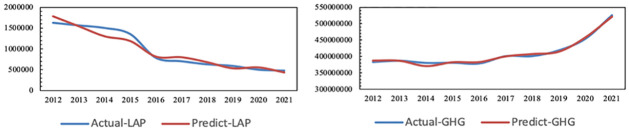
Actual and simulated emissions of air pollution and greenhouse gases in Zhejiang from 2012 to 2021.

It is concluded that the adjusted R^2^ of air pollution is 0.897, F = 20.052, p = 0.006<0.05 and the adjusted R^2^ of greenhouse gas is 0.968, F = 71.545, p = 0.001<0.05. Therefore, the prediction model of air pollution and carbon emissions have high precision and can be used for prediction and analysis. Additionally, the results of actual and simulated emissions of air pollution and greenhouse gas are shown in Fig 3.

#### 4.1.4 Forecast model in Anhui

Based on Formula (8) and the data from 2012 to 2021, the prediction model of air pollution and carbon emissions in Anhui Province is constructed as follows by using partial least square method.


$$E_{GHG}=EXP\;(276.969-29.998\;ln\;P+1.07\;ln\;A+0.654\;ln\;UR-0.004\;ln\;IS+0.332\;ln\;T)$$ (15)

$$E_{LAP}=EXP\;\;(451.669-47.214\;ln\;P+0.666\;ln\;A-6.628\;ln\;UR-0.626\;ln\;IS-1.34\;ln\;T)$$ (16)

According to the forecast model, with other variables held constant, air pollutant emission in Anhui is directly proportional to the economic scale, for every 1% increase in energy intensity, air pollutant emission will be increased by a mean of 0.666%, and inversely proportional to other factors, for every 1% increase in population size, urbanization level, industrialization level and energy intensity, air pollutant emissions will be decreased by a mean of 47.214%, 6.628%, 0.626% and 1.34%, respectively.

The carbon emissions in Anhui is directly proportional to the economic scale, urbanization level and energy intensity, for every 1% increase in economic scale, urbanization level and energy intensity, carbon emissions will be increased by a mean of 1.07%, 0.654% and 0.332% respectively, and inversely proportional to the population size and industrialization level, for every 1% increase in population size and industrialization level, carbon emissions will be decreased by a mean of 29.998% and 0.004%.

**Fig 4:**
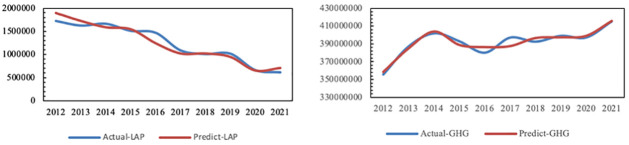
Actual and simulated emissions of air pollution and greenhouse gases in Anhui from 2012 to 2021.

It is concluded that the adjusted R^2^ of air pollution is 0.871, F = 96.989, p = 0.000<0.05 and the adjusted R^2^ of greenhouse gas is 0.816, F = 33.086, p = 0.002<0.05. Therefore, the prediction model of air pollution and carbon emissions has high precision and can be used for prediction and analysis. Additionally, the results of actual and simulated emissions of air pollution and greenhouse gas are shown in Fig 4.

### 4.2 Scenario analysis of synergistic emission equivalent

#### 4.2.1 Scenario forecast results of synergistic emission equivalent

**Fig 5:**
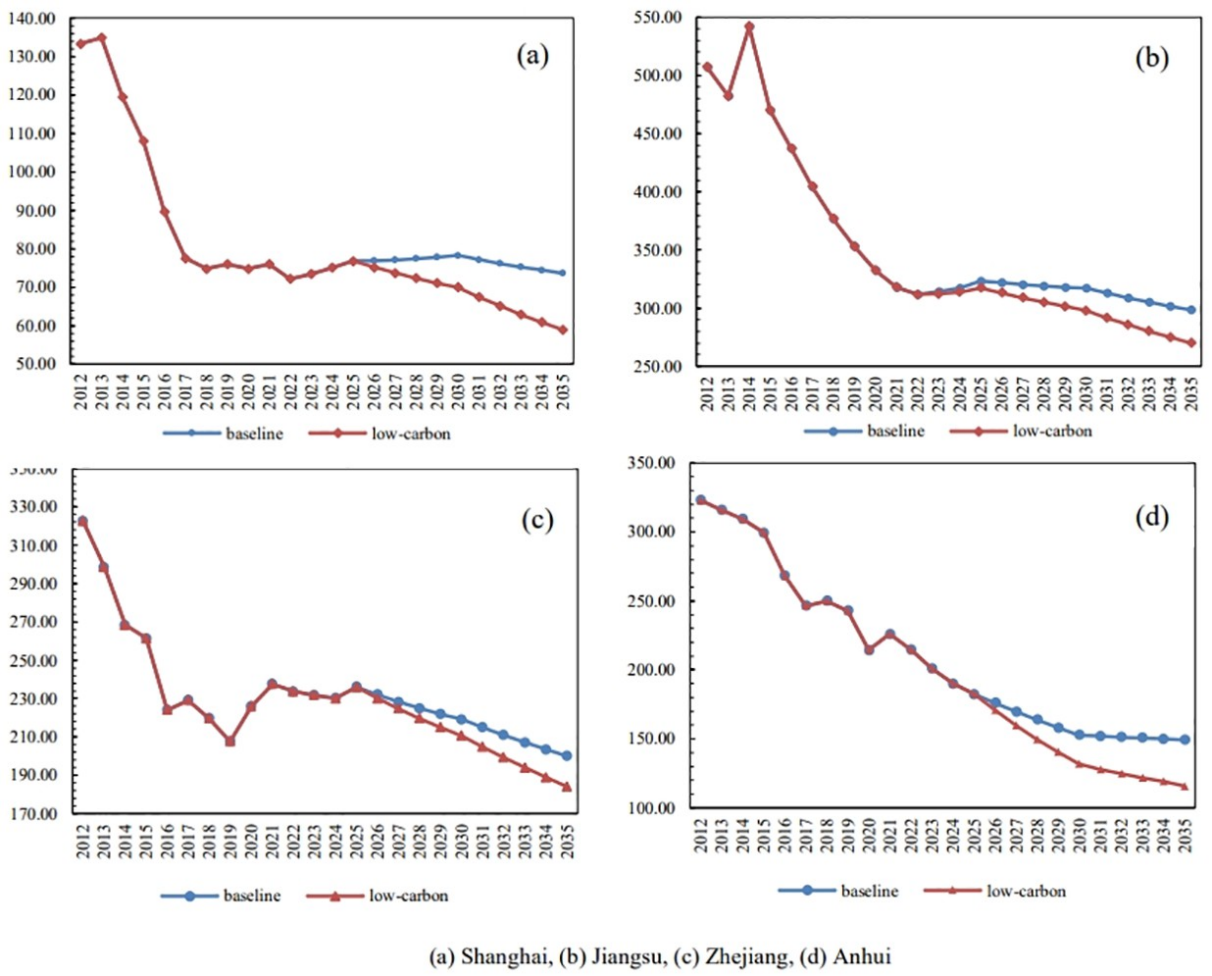
Synergistic emission equivalent of different scenarios from 2012 to 2035 /10,000 tons. (a) Shanghai, (b) Jiangsu, (c) Zhejiang, (d) Anhui.

According to Formula (4) and the parameters set out, the synergistic emission equivalent of three provinces and one city is calculated and the synergistic emission equivalent in 2012–2035 is calculated and estimated in different scenarios. The results are shown in Fig 5.

#### 4.2.2 Analysis on synergistic emission equivalent

(a) The synergistic emission equivalent of Shanghai City has been declining year by year since 2013, and the downward trend has gradually slowed down after 2017. From 2017 to 2025, there is a certain degree of fluctuation. From 2025, under the baseline scenario, the synergistic emission equivalent still continues to rise slightly until 2030, however, that will keep a considerable downward trend under the low-carbon scenario.
(b) The change trend of emissions under the baseline and low-carbon scenarios is relatively consistent, showing a constant declining since 2015 and slightly rebound from 2022 to 2025, then both decline year by year from 2025. The ratio under the low-carbon scenario is greater than that under the baseline scenario.
(c) The synergistic emission equivalent of Zhejiang Province has decreased year by year since 2012 and experienced a nine years’ fluctuation from 2016 to 2025, then it will decline in a relatively high rate under both scenarios.
(d) The synergistic emission equivalent of Anhui Province shows a constant and relatively smooth trend since 2012 under both scenarios and the rate of declining will decrease from 2030.

To sum up, comparing four trends shown in Fig 5, although little increase or decrease occur in certain years, the overall performance of synergistic emission equivalent in Yangtze River Delta is consistent.

### 4.3 Scenario analysis of synergy coefficient

#### 4.3.1 Scenario forecast results of synergy coefficient

Based on the calculated synergistic emission equivalent, the synergy coefficients of three provinces and one city in 2013–2021 are calculated according to formula (4) and the synergy coefficients in 2026–2035 are estimated by scenarios. The results are shown in Table 6.

**Table 6:** Scenario forecast of regional synergy coefficient in Yangtze River Delta from 2026 to 2035.

	Baseline Scenario				Low-carbon Scenario			
	Shanghai	Jiangsu	Zhejiang	Anhui	Shanghai	Jiangsu	Zhejiang	Anhui
2026	-6.549	-7.819	28.875	2.752	17.701	19.135	9.138	3.283
2027	-6.549	-7.819	28.875	2.752	17.701	19.135	9.138	3.283
2028	-6.549	-7.819	28.875	2.752	17.701	19.135	9.138	3.283
2029	-6.549	-7.819	28.875	2.752	17.701	19.135	9.138	3.283
2030	-6.587	-7.613	35.183	2.749	16.768	18.671	8.869	3.306
2031	22.278	19.135	9.138	3.592	5.331	7.844	5.153	20.931
2032	22.278	19.135	9.138	4.965	5.331	7.844	5.153	20.931
2033	22.278	19.135	9.138	4.965	5.331	7.844	5.153	20.931
2034	22.278	19.135	9.138	4.965	5.331	7.844	5.153	20.931
2035	20.798	18.671	8.741	5.041	5.371	7.613	5.194	21.585

#### 4.3.2 Analysis of synergy coefficient

Under the Baseline Scenario, the performance of synergy coefficient in Shanghai City and Jiangsu Province are similar, both show negative during 2026 and 2030 which means incompatible emissions, however, 2030 is the turning point after which the synergy coefficient in both cities turns to be positive and exceed 1.2 which illustrates air pollution and carbon emissions achieve synergistic reduction and the reduction rate of air pollution is higher than carbon emissions. Besides, the synergy coefficients of Zhejiang and Anhui Province always show a positive value which is over 1.2 between 2026 and 2035, and that means both air pollution and carbon emissions reduce in that period and the rate of air pollution reduction is higher than that of carbon reduction.

Under the Low-Carbon Scenario, synergy coefficient value in four provinces and cities are all positive (between 2.749 and 19.135) and exceed 1.2 which means the whole Yangtze River Delta region could achieve synergistic reduction between the period of 2026–2035 under this scenario and the rate of air pollution reduction is higher than that of carbon reduction. In addition, the changing trend of that in Anhui Province is different from others which shows a constant upwarding trend.

## 5. Discussion

1. Consider the synergistic emission equivalent and synergy coefficient comprehensively when evaluating the synergistic effect of air pollution control and carbon reduction in the Yangtze River Delta region as the total reduction of synergistic emission equivalent does not mean synergistic emission reduction. Taking Zhejiang Province and Jiangsu Province under the baseline scenario as an example, although both provinces’ synergistic emission equivalent shows a continuous decline trend from 2026 to 2035, the latter does not achieve synergistic emission reduction during this period as analysed in 3.3.2, while the former does the opposite. Based on the analysis of Formula (3), it is the difference in the contribution of air pollution and carbon emissions to the synergistic emission equivalent that leads to this result.
2. In general, the change trend of synergistic emission equivalent of the three provinces and one city in the Yangtze River Delta region from 2012 to 2035 is relatively consistent, but Shanghai and Zhejiang Province show varying degrees of fluctuation between 2017 and 2025. According to the analysis in this paper, due to the fluctuation of the international situation and the impact of COVID-19, especially the impact of the Sino-US trade war, the consumption of energy and resources in the Yangtze River Delta region increased slightly to mitigation the weak economic growth, resulting in the increase of carbon emissions. Meanwhile, the air pollution reduction has gradually slowed down since 2017, with an average annual reduction rate of about half of that before 2017, showing air pollution reduction has entered the bottleneck stage. Therefore, the overall cooperative emission equivalent fluctuates from Fig 5. However, the 14th Five-Year Plan period is a critical period for China to promote the co-benefits of pollution control and carbon reduction and it is of great significance for Yangtze River Delta region to strictly implement the goals and requirements of the 14th Five-Year Plan and after the plateau period, three province and on city will achieve annual emission reduction successively between 2025 and 2030. Therefore, this paper holds the view that the Yangtze River Delta region meets the conditions to achieve the carbon peak before 2030.
3. limitations and uncertainties. First of all, the equivalent coefficient in Table 1 calculated in this paper are based on the whole country, which may be different from the Yangtze River Delta region. Secondly, the forecast model in this paper is based on the past 10-year data from 2012 to 2021, however, China is in a stage of rapid development, past data are uncertain when predicting future trends. Thirdly, the scenario prediction in this paper is mainly based on relevant government documents, as mentioned in the second point, China is developing so rapidly that it is difficult to set precisely, so this paper prefers qualitative conclusions rather than quantitative.

## 6. Conclusions

### 6.1 Conclusions

Based on the development reality of the Yangtze River Delta, the main economic and social development indicators of the 14^th^ Five-Year Plan and previous research results [30, 31], selected population size, economic scale, industrialization level, urbanization level, and energy intensity as the influencing factors and constructed an extended STIRPAT model for air pollution control and carbon reduction in the Yangtze River Delta; On this basis, the forecast analysis is further carried out from the two dimensions of synergistic emission equivalent and synergy coefficient. The following conclusions are drawn:

1. Based on the STIRPAT model, the economic scale and energy intensity of the three provinces and one city are directly proportional to carbon emissions, while the impact of other factors on carbon emissions shows spatial heterogeneity. The impact of all influencing factors on air pollution emissions depends on the specific conditions of each province and city.
2. In terms of synergistic emission equivalent, the overall trend of synergistic emission equivalent of three provinces and one city is relatively consistent, even though there are different degrees of fluctuations from 2017 to 2025. In addition, after different lengths of plateau period, Yangtze River Delta region meets the condition to enter a new stage of synergistic emission reduction by 2030 at the latest.
3. In terms of synergy coefficient, under each scenario, air pollution and carbon emissions will achieve synergistic reduction from 2031 to 2035, and the synergy coefficient is between 2.749 and 19.135, both of which are greater than 1.2, indicating that air pollution and carbon emissions will decrease simultaneously, and the rate of air pollution reduction will be higher than that of carbon reduction. Yangtze River Delta region meets the the conditions to enter the stage of pollution control and carbon reduction not later than 2030.

### 6.2 Recommendations

1. The key to promoting synergy between pollution control and carbon emission reduction in the Yangtze River Delta is to strictly control the total amount of carbon emissions; the faster the low-carbon process, the greater the carbon emission reduction, and the more significant the synergistic benefits of pollution and carbon reduction. Therefore, the government can improve the top-level design by combining policies related to air pollution control with measures related to carbon emission reduction, so as to realize synergistic governance between the two. At the same time, the Government should strengthen its support and guidance for enterprises to carry out technological innovation in energy conservation and emission reduction, change the mode of economic development, promote the transformation and upgrading of the industrial structure, and vigorously develop a low-carbon, recycling, and green economy, so as to further promote carbon emission reduction.
2. The Yangtze River Delta region is densely populated, and the total population is on an upward trend, so the impact of population growth on the increase in carbon emissions is unlikely to be rapidly reduced in a short period of time. However, the government can promote and publicize green living, actively guide the public to choose a low carbon lifestyle and enhance people’s awareness of environmental protection in order to reduce carbon emissions at the individual level, thereby achieving a positive impact on the synergistic benefits of pollution control and carbon reduction in the medium and long term.
3. There are significant differences in resource endowment, technological development, and geographic location among regions, so based on a full understanding of the social, economic and technological development of each province and city, we should promote pollution reduction and carbon reduction policies according to local conditions. For example, Anhui Province is relatively weak in science and technology compared with Shanghai and other regions, so it should focus on the introduction and development of green technology and strengthen the innovation of energy-saving and emission reduction technology, so as to further realize the reduction of pollution and carbon emissions.

## References

[1] Zhang H B. Reflections on Several Issues of Global Climate Governance. [J].Journal of Huazhong University of Science and Technology(Social Science Edition),2022.05.05.

[2] Ayres R U, Walter J. The greenhouse effect: Damages, costs, and abatement [J]. Environmental and Resource Economics, 1991, 1(3):237–270.

[3] IPCC (2001). IPCC Third Assessment Report Climate Change 2001[J]. The Scientific Basis-WG I SPM, 2001, 2001.

[4] Mao X Q, Zeng A, Xing Y K, et al. From concept to action: a review of research on co-benefits and co-control of greenhouse gases and local air pollution reductions[J]. Climate Change Research,2021,17 (3):255–267.

[5] Monge Juan J.; McDonald Garry W. Assessing the economic implications of national climate change mitigation policies on cities: A CGE analysis of Auckland, New Zealand [J]. Journal of Cleaner Production, 2023, 418.

[6] Oh Inha and Yoo Wang-Jin and Yoo Yiseon. Impact and Interactions of Policies for Mitigation of Air pollution and Greenhouse Gas Emissions in Korea[J]. International Journal of Environmental Research and Public Health, 2019, 16(7): 1161–1161. 30935125 10.3390/ijerph16071161 PMC6479864

[7] Nam Kyung-Min et al. Synergy between pollution and carbon emissions control: Comparing China and the United States[J]. Energy Economics, 2014, 46: 186–201.

[8] Yu S, Zhang S, Zhang Z J, et al. Scenario Simulation and Effects Assessment of Co-control on Pollution and Carbon Emissions Reduction in Beijin. [J/OL]. Environmental Science,2022,1–17.

[9] Liu M H, Yue Y Y, Liu S N, et al. Multi-dimensional Analysis of the Synergistic Effect of Pollution Reduction and Carbon Reduction in Tianjin Based on the STIRPAT Model [J/OL].Environmental Science,2022,1–13. 10.13227/j.hjkx.202204086 36922189

[10] Sun Y M, Zhou C Y. The spatio-temporal evolution characteristics and influencing factors of collaborative governance of air pollution in the Yangtze River Delta region. [J]. Geographical Research,2022,41(10):2742–2759.

[11] Zhang Y, Sun Q, Xue J J, Yang C H. Synergistic effects of pollution control and carbon reduction and their pathways. [J]. China Population, Resources and Environment,2022,32(05):1–13.

[12] Asjad Naqvi. Decoupling trends of emissions across EU regions and the role of environmental policies[J]. Journal of Cleaner Production, 2021, 323.

[13] Portugal-Pereira Joana et al. Interactions between global climate change strategies and local air pollution: lessons learnt from the expansion of the power sector in Brazil[J]. Climatic Change, 2018, 148(1–2): 293–309.

[14] Yuan X L, Xi J H, Zhong C C, et al. Collaborative driving factors and realization paths of "pollution and carbon reduction" in Chinese cities [J]. Journal of Management,2023,36(04):26–46.

[15] Zhang X C, Cao X, Song L H. Research on measurement and influencing factors of pollution and carbon reduction efficiency in China: based on super efficiency SBM-Tobit model [J]. Ecological Economics,2023,39(10):174–183.

[16] Wang F T, Fang K, Yu C. Decoupling Between Industrial Energy-related Carbon Emissions and Economic Growth and Its Driving Factors in Beijing, Tianjin, and Hebei Urban Agglomeration–Empirical Study Based on Tapio Decoupling and LMDI Mode l. [J].Journal of Industrial Technological Economics,2019,38(08):32–40.

[17] Wang L., Li P., Yu S. et al. Predicted impact of thermal power generation emission control measures in the Beijing-Tianjin-Hebei region on air pollution over Beijing, China. Sci Rep,2018,8,934 doi: 10.1038/s41598-018-19481-0 29343860 PMC5772530

[18] Zhai H X, Yue Y Y, Liu S N, et al. Analysis of the synergistic effect of pollution reduction and carbon reduction in Tianjin’s”Blue Sky Defense Battle” [C].Proceedings of the 2022 Science and Technology Annual Conference of the Chinese Society of Environmental Sciences (1)2022.016183.

[19] Adila ALIMUJIANG, Jiang P, Dong H J, et al. Synergy and co-benefits of reducing CO2 and air pollutant emissions by promoting new energy vehicles: A case of Shanghai[J]. Acta Scientiae Circumstantiate, 2020,40(5): 1873–1883.

[20] Wang M, Feng X Z, Du X L et al. Evaluation of co-controlling GHGs from pollutant reduction facilities in the industrial sectors, empirical analysis based on data in Chongqing city [J]. Climate Change Research, 2021, 17(3): 296–304.

[21] Wang J N, Yan G, Lei Y. Coordinate to promote pollution reduction and carbon reduction to help achieve the construction of a beautiful China and the goal of”dual carbon”,2022.

[22] He K B. Technical Manual for compiling emission inventory of Urban Air Pollution Sources [R]. Beijing: Tsinghua University, 2018.

[23] Shan Y L, Liu J H, Liu Z, et al. New provincial CO_2_ emission inventories in China based on apparent energy consumption data and updated emission factors.[J] Applied Energy,2016,184:742–750.

[24] Gao Y B, Xing Y K, He F, et al. Research on co-control effectiveness evaluation of energy saving and emission reduction measures in China’s iron and steel industry [J]. Climate Change Research, 2021, 17 (4): 388–399.

[25] Sun R. Improving Tapio Decoupling Measurement Method and its Applications,2014(08):7–11.

[26] Tapio P. Towards A Theory of Decoupling: Degrees of Decoupling in the EU and the Case of Road Traffic in Finland Between 1970 and 2001 [J]. Transport Policy,2005,12(2):137 151.

[27] Zhou X, Zhang M, Zhou M, et al. A Comparative Study on De- coupling Relationship and Influence Factors Between China’s Regional Economic Development and Industrial Energy—related Carbon Emissions [J]. Journal of Cleaner Production,2017,142:783 800.

[28] Ehrlich P R, Ehrlich A H. Population, resources, environment: issues in human ecology [M]. San Francisco:Freeman,1970:89–157.

[29] Dietz T, Rosa E A. Rethinking the environmental impacts of population, affluence, and technology [J].Human EcologyReview,1994(1):277–300.

[30] Jiang H Q et al. Influencing factors and countermeasures of carbon peaking in Yangtze River Delta urban agglomeration from the heterogeneity perspective. [J].Urban Problems,2022(08):52–61.

[31] Li Z Q, Wu Z Q, Wu H Y. Zero carbon Evaluation of Cities in the Yangtze River Delta. [J].Fudan Journal(Social Sciences Edition),2022,64(05):142–151.

[32] Zhang Z, Ren Y M, Dong H J. Research on carbon emission peaking and low-carbon development of cities: a case of Shanghai. [J].Environmental Engineering, 2020, 38(11): 12–18.

